# Safety and Efficacy of the C-117 Formula for Vulnerable Carotid Artery Plaques (Spchim): A Randomized Double-Blind Controlled Pilot Study

**DOI:** 10.1155/2019/9746492

**Published:** 2019-07-17

**Authors:** Baoying Gong, Xiuyan Chen, Rongming Lin, Feng Zhang, Jingxin Zhong, Qixin Zhang, Yuexiang Zhou, Haijun Li, Liling Zeng, Zonghua Jiang, Jianwen Guo

**Affiliations:** ^1^Guangzhou University of Chinese Medicine, No. 232, Waihuan East Road, Guangzhou Higher Education Mega Center, Panyu District, Guangzhou, Guangdong Province 510006, China; ^2^Guangdong Second Traditional Chinese Medicine Hospital, 60 Hengfu Road, Yuexiu District, Guangzhou, Guangdong Province 510095, China; ^3^The 6th Affiliated Hospital of Sun Yat-Sen University, 26 Erheng Road, Yuancun, Tianhe District, Guangzhou, Guangdong Province 510655, China; ^4^The 2nd Teaching Hospital of Guangzhou University of Chinese Medicine (Guangdong Provincial Hospital of Traditional Chinese Medicine), 111 Da'de Road, Yuexiu District, Guangzhou, Guangdong Province 510120, China; ^5^People's Hospital of Ganzhou City, 17 Hongqi Avenue, Ganzhou City, Jiangxi Province, 341000, China

## Abstract

**Objective:**

To investigate the safety and efficacy of the Herbal Medicine C-117 (C-117) formula in the treatment of carotid atherosclerotic vulnerable plaques.

**Methods:**

This was a prospective, single-centre, randomized, double-blind study. A total of 120 eligible patients were randomly divided into two groups to receive the C-117 formula or placebo. As the basic treatment, both groups were treated according to the* Guidelines for Secondary Prevention of Ischemic Stroke/Transient Ischemic Stroke in China* using statins to regulate blood lipids, blood pressure lowering drugs, drugs for controlling blood sugar, and antiplatelet drugs according to the indications. The primary outcomes were the change in stability, the mean change of the plaque Crouse score, and the area and number of bilateral carotid artery plaques before and after 6 months of treatment. The secondary outcomes were the total number of cardiocerebrovascular events during the treatment and follow-up and the mean changes of lipid levels.

**Result:**

After 180 days of treatment, the plaque Crouse score(95% CI, 0.39 (0.01-0.77), P=0.046) and plaque area (95% CI, 2.14 (-10.10-14.39), P=0.727) were lower in the C-117 formula group than that before treatment. The plaque Crouse score of the control group (95% CI, 0.17 (-0.24-0.57), P=0.417) was lower than that before treatment, while the plaque area (95% CI, -0.35 (-9.35-8.65), P=0.938) increased, but without statistical significance. There was no significant difference in the reduction of the intima-media thickness (IMT), plaque Crouse score, or plaque area between the two groups after treatment (P>0.05). Subgroup analysis of patients whose Lipitor medication time ≥ 20% of the 6-month treatment showed that the levels of total cholesterol, triglycerides, and low-density lipoprotein were lower in the two groups after treatment than before, and the low-density lipoprotein levels in the C-117 formula group significantly decreased (95% CI, 2.99 (-0.08-0.39), P=0.005), but there was no statistical difference between the two groups after treatment (P>0.05). No serious adverse events occurred in the two groups after 180 days of treatment.

**Conclusion:**

The C-117 formula may be antiatherosclerotic by strengthening statins to reduce the low-density lipoprotein levels and reducing the carotid plaque Crouse scores. Clinical trials with large sample sizes, long-term interventions, and follow-up are needed to investigate the efficacy of the C-117 formula.

**Clinical Trials Registration:**

This trial is registered with clinicaltrials.gov identifier: NCT03072225 (registered retrospectively on 1st March 2017).

## 1. Introduction

Studies have shown that, between 1990 and 2016, global stroke mortality and morbidity gradually declined, but the morbidity in China was still rising and the burden of stroke as worse than before [1]. Stroke is still the leading cause of death and disability in China, and ischemic stroke's incidence rates is the highest among pathological types of stroke [2, 3]. Rupture of carotid atherosclerosis (CAS) plaques is an important cause of ischemic stroke. Studies have shown that, among Chinese symptomatic patients, there were more people with vulnerable carotid artery plaques than those with carotid stenosis (≥50%) [4].

At present, the treatment of CAS plaques mainly includes tertiary drug therapy (antiplatelet, lipid lowering, and blood pressure control) and surgical treatment (carotid endarterectomy; carotid artery stenting) [5, 6]. Vascular stent implantation directly covers unstable plaques, and there is a risk of restenosis after vascular stent surgery, while carotid endarterectomy has not been widely available in China. Statins, the most commonly used lipid-lowering drugs also have shortcomings. The SPARCL study [7] showed that high-dose atorvastatin calcium tablets (80 mg) only reduced the stroke recurrence rate by 16% over 5 years compared with placebo but increased the risk of haemorrhagic stroke. Statins also have other side effects, such as liver damage, muscle damage, and so on [6, 8, 9]. Therefore, there is still a lack of safe and effective drugs for treating CAS-vulnerable plaques.

Traditional Chinese Medicine is widely used as a secondary prevention in various types of chronic diseases in China, including atherosclerosis (AS). In the theory of traditional Chinese Medicine, the plaque is considered to be a tangible pathological factor (tangible evil) that is a pathological product of phlegm and blood stasis. The C-117 formula has been patented (Chinese Patent, application no. 201610326186.9) and consists of four kinds of herbal and insect medicine (*Hirudo *(ShuiZhi),* Atractylodis Rhizoma *(CangZhu),* Curcumae Zedoary *(EZhu), and* Endothelium Corneum Gigeriae Galli *(JiNeiJin)), which might remove blood stasis and clear phlegm, making the meridian smooth again. Traditional Chinese Medicine theory believes that animal medicine has stronger pharmacological activity than botanical medicine.* Hirudo* and* Endothelium Corneum Gigeriae Galli* are animal medicines that have the effect of removing a disease from the deep meridians and making the meridians smooth. Modern pharmacology believes that they can anticoagulation, inhibit endothelial cell proliferation, and antioxidation, and regulate blood lipids [10–13]. Years of clinical practical experience has shown that the Herbal Medicine C-117 formula might stabilize plaques or reduce the area of vulnerable plaques. The preexperiment, a small sample size and nonrandomized controlled study, showed that compared to treatment with atorvastatin calcium tablets alone, combination therapy with the C-117 formula was more effective in reducing the carotid IMT (F=30.806, P≤0.001), plaque Crouse score (F=11.815, P=0.001), and plaque area [14].

However, there is still a lack of well-designed clinical trial evidence. Therefore, we designed a single-centre, prospective, randomized, double-blind, and placebo-controlled clinical trial to evaluate the safety and efficacy of the C-117 formula.

## 2. Methods

### 2.1. Setting and Study Population

This study was a single-centre, prospective, randomized, double-blind and placebo-controlled clinical pilot trial with two parallel groups. The study received ethical approval from the Ethics Committee of Guangdong Provincial Hospital of Chinese Medicine (Ethical Review no. Y2016-001) and was registered with ClinicalTrials.gov (ClinicalTrials.gov ID: NCT03072225).

All participants were recruited by posters in the hospital or in the hospital's WeChat official account ads. All patients in the screening session were informed of the protocol and signed a consent form. A total of 120 eligible patients who had hyperlipidaemia and vulnerable carotid plaques (Table 1 shows the detailed eligibility and exclusion criteria) were randomly divided into the C-117 formula group and the placebo group according to a 1:1 ratio and received a 6-month treatment.

### 2.2. Randomization and Blinding

The participants were assigned at random to one of the two treatment groups at a ratio of 1:1. Block randomization is generated and stratified by the PROC PLAN process using the SAS software version 9.2. The stochastic allocation procedure was saved in the Key Unit of Methodology in Clinical Research of Guangdong Province Hospital of Traditional Chinese Medicine (KYMCRGOHTCM). Stratified block randomization was concealed using a sequentially numbered and opaque envelope.

Eligible patients were randomized into the C-117 formula group or the placebo group by obtaining medicines associated with the given drug codes in accordance with the order of visits. Participants, investigators, statisticians, and all study staff were blinded. Only the data administrators were permitted access to the unblinded data.

### 2.3. Interventions

All eligible patients received the basic treatment according to the* Guidelines for Secondary Prevention of Ischemic Stroke/Transient Ischemic Stroke in China 2014* [15], including treatment of basic diseases (control of hypertension, diabetes, hyperlipidaemia, and other diseases) and establishment of a good lifestyle.

For the C-117 group, the C-117 formula is composed of 4 herbal and insect medicines,* Hirudo *(ShuiZhi),* Atractylodis Rhizoma *(CangZhu),* Curcumae Zedoary *(EZhu), and* Endothelium Corneum Gigeriae Galli *(JiNeiJin). For the placebo group, the placebo is composed of dextrin, powdered sugar, starch, and liquid caramel. Its colour, smell and form are consistent with the C-117 formula. Both C-117 and the placebo drugs were prepared by the Kangyuan Pharmaceutical Factory according to Good Manufacturing Practices (GMP). Each pill had a diameter of 4.2 mm and was sealed and packaged in an opaque plastic bag (6 g per bag). Quality control was strictly enforced throughout the trial.

Patients were instructed to take the pills one bag each time, twice a day for 6 months. Only one month's dose could be obtained at a time, so each patient had to return to the hospital once a month to receive the drug from the investigators. The investigators were responsible for dispensing the medication and recording the adverse events that occurred during the medication.

### 2.4. Measurements

The primary outcome measures were the change in stability, the mean change of the plaque Crouse score, and the area and number of bilateral carotid artery plaques before and after 6 months of treatment.

A carotid artery ultrasound was used to the detect carotid artery IMT at the bilateral common carotid artery, bilateral internal carotid artery, bilateral external carotid artery, and its bifurcation 1.0 cm proximal and 1.0 cm distal to the carotid, and the average of the three locations was taken as the carotid IMT. Carotid artery IMT≥1.0 mm is defined as intimal thickening and localized IMT ≥1.5 mm as defined as a plaque [16].

Stability was defined according to the description of the colour ultrasound result: homogeneous hypoechoic plaques and irregular and ulcerated plaques represented unstable plaques, while homogeneous echogenic plaques and strong echogenic plaques represented stable plaques. The sum of the maximum thickness of each plaque in each blood vessel is defined as the plaque Crouse score for that blood vessel, and the sum of the bilateral carotid artery Crouse score was defined as the plaques Crouse score for each patient. To determine the plaque area, 3 diameters of each plaque were measured, and the two largest diameter lines were selected as the length-width multiplication. The sum of the areas of both carotid plaques was the total plaque area of the patient. The number of plaques was the sum of the number of bilateral carotid plaques.

The secondary outcomes were as follows: (1) The total number of cardiocerebrovascular events within 12 months (6 months treatment period and 6 months follow-up): cardiovascular and cerebrovascular events refer to events that cause vascular atherosclerosis and lead to organ lesions, such as coronary heart disease and stroke; (2) the mean changes of lipid levels (including triglyceride, cholesterol, low-density lipoprotein, and high density lipoprotein) before and after the 6-month treatment; (3) the mean changes of bilirubin levels (including the total bilirubin, direct bilirubin, and indirect bilirubin) before and after the 6-month treatment.

All adverse symptoms or adverse events were recorded throughout the study.

### 2.5. Sample Size

According to the study by Hirayama, A, et al. [17], the most commonly used drug for treatment of hyperlipidaemia, Atorvastatin, could decrease the volume of plaques by 8.7%. We supposed that the basic treatment combined with the C-117 formula could decrease it by 15%. According to this supposition and as calculated by the PASS 11.0 software, a sample size of 210 in each group achieved a 90% power and ruled out a two-sided type I error of 5% to detect a superiority margin difference of 15% in this trial. Considering a 15% loss to follow-up, the total sample size was adjusted to 247 in each group. Our preexperiment enrolled 103 participants, and the results showed that the C-117 formula might stabilize the vulnerable plaques [14], but it was a nonrandomized controlled study that reduced the credibility of the results. This study was designed as a randomized controlled and pilot study, so we reduced the sample size to 120 patients, with 60 patients in each group.

### 2.6. Statistical Analysis

All the statistical analyses were performed with the Statistical Package for Social Science statistics (SPSS 18.0). The statistical analysis set included Full-Analysis Set, Per-Protocol Population Set, and Safety Set. According to the principle of intention-to-treat analysis, FAS was a randomized group with at least one intervention and included postintervention evaluation data; PPS was in accordance with the inclusion criteria specified in the trial protocol, and compliance was greater than 80%; the outcome of the treatment was complete and there was no outcome evaluation after the last treatment. Drugs or treatments that might affect the efficacy evaluation were not used during the trial. The PPS dataset is the secondary dataset for the efficacy evaluation of this study; SS was all randomized. Grouping was used as long as patients underwent a study treatment and had performed at least one safety assessment. For FAS, this study used the following data-filling method: in the case of 0-180 days of detachment, withdrawal (nondeath): LOCF (last carry-over).

Demographic and baseline data were analyzed with standard, descriptive statistics. The blood lipid levels, number of plaques, plaque area, Crouse score, and bilirubin content are expressed as the mean ± standard deviation. The total cholesterol content, plaque Crouse score, total bilirubin, and indirect bilirubin content were compared before and after treatment with paired design t test. Triglyceride and low-density lipoprotein levels were compared before and after treatment. The independent sample rank sum test was used to compare the block area and posttreatment groups. The statistical description of the qualitative variables included the number of cases per group, the composition ratio (rate), and the chi-square test. All statistical tests were performed by a two-sided test. The test level was defined as *α*=0.05, and the difference was statistically significant at P<0.05. All statistical analyses were performed on blinded group allocations.

## 3. Result

**Figure 1:**
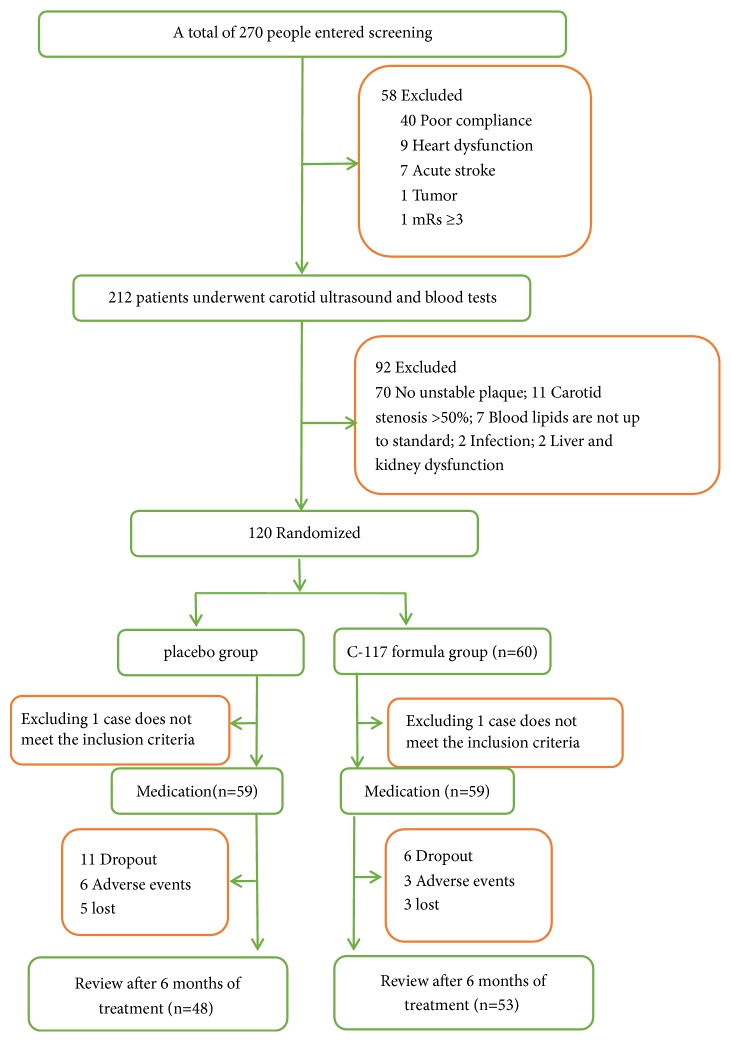
Flowchart of the participants through the trial.

From March 1st, 2017, to September 18th, 2018, a total of 270 people entered screening, and 120 cases were included in the study. One case from each group did not meet the inclusion criteria and was excluded. Therefore, a total of 118 patients were treated with medication (see Figure 1), of which 56 were male. More than half of the subjects in the test population were ≥ 60 years old (89 (75.42%), 56.78% of the subjects had hypertension, and 45.76% had diabetes. Of the patients, 79.7% took Lipitor (Atorvastatin calcium tablets 20 mg qn) as a lipid-lowering treatment, and only 4 people did not take lipid-lowering drugs. After 6 months, a total of 17 subjects did not complete the trial (6 in the C-117 formula group and 11 in the placebo group). The two groups were baseline balanced and comparable (Table 2).

### 3.1. Plaque Characteristics

In the C-117 formula group after 6 months of treatment, the plaque Crouse scores (95% CI, 0.39 (0.01-0.77), P=0.046<0.05) and plaque area (95% CI, 2.14 (-10.10-14.39)), P=0.727) were lower than before treatment, and the change in the plaque Crouse score was statistically significant. The plaque Crouse score of the control group (95% CI, 0.17 (-0.24-0.57), P=0.417) was lower than that before treatment, and the plaque area (95% CI, -0.35 (-9.35-8.65), P=0.938) increased compared with the area pretreatment, though neither of these were statistically significant (Table 3). There was no significant difference in the reduction of IMT, plaque Crouse score, or plaque area between the two groups after treatment (P>0.05).

### 3.2. Blood Lipid and Bilirubin Changes

To reduce interference, we only analysed lipids levels in patients who were treated with Lipitor. After treatment, the triglycerides and total cholesterol of the two groups were lower than before, and the total cholesterol in the C-117 formula group significantly decreased (P<0.05) (Table 4). There was no significant difference in blood lipids between the two groups after treatment (P>0.05). After sorting out and analyzing the database again, we found that some patients took lipid-lowering drugs only for a very short time, which may cause inaccuracy in the results of analysis. So we divided the patients who had not taken or the lipid-lowering medication time < 20% of the 6-month treatment into one group, a total of 16 people (Table 5). After removing this group of patients, we analyzed the blood lipid levels before and after treatment in patients taking Lipitor again. It showed that the triglyceride, total cholesterol, and low-density lipoprotein (LDL) were found to be reduced in both groups, and the triglyceride change in the control group and the low-density lipoprotein change in the C-117 formula group were statistically significant. There was no statistical difference between two groups after treatment (Table 6).

After treatment, the total bilirubin and direct bilirubin increased in both groups, and the direct bilirubin significantly increased in both groups (P<0.05) (Table 7). There was no statistically significant difference (P>0.05) when comparing bilirubin between the two groups after treatment (Table 7).

### 3.3. Adverse Events

During the 6-month treatment period, there were 9 cases of a blood pressure drop in the two groups (5 in the C-117 formula group and 4 in the placebo group). There were 2 cases of constipation and 3 cases of diarrhoea, all of which were from the placebo group. There were 9 other adverse events (2 in the C-117 formula group and 7 in the placebo group). A total of 9 patients developed a decrease in blood pressure, and blood pressure returned to normal after the antihypertensive drug was stopped. The blood pressure drop occurred between June and August, which may be related to vasodilation in summer. We think the blood pressure drop was independent of the drug. In the control group, constipation could be alleviated after stopping the drug and may have been related to the drug. Diarrhoea, dizziness, acute cholecystitis, tonsillitis, palpitations, and hyperhidrosis were considered to be unrelated to the test drug. Six patients in the placebo group withdrew from the trial due to adverse events, and 5 were lost to follow-up. Three patients in the C-117 formula group withdrew due to adverse events, and 3 patients were lost to follow-up.

## 4. Discussion

This study aimed to investigate the safety and efficacy of the C-117 formula in the treatment of CAS-vulnerable plaques. After 180 days of medication, the plaque Crouse scores of the two groups were lower than before and the total cholesterol and blood lipids decreased compared with those before treatment. The change of the C-117 formula group was statistically significant, but no significant differences were found when comparing between groups after treatment. Although adverse events occurred during the 6-month treatment, there was no liver or kidney dysfunction, and all adverse events were associated with a weak correlation with the C-117 formula. We believe that the C-117 formula provided better results.

Studies have found that the serum bilirubin levels in patients with AS are negatively correlated with the TIM thickness. The serum bilirubin levels is one of the risk factors for atherosclerosis. Although all molecular mechanisms behind its biological effects are still unknown, bilirubin is an important prognostic marker and a potential therapeutic target [18–20]. Therefore, we also compared the bilirubin levels of the two groups and found that the total bilirubin and direct bilirubin levels in the two groups were higher than those before treatment, but there was no difference between the two groups after treatment. Statins, the most commonly used lipid-lowering drug, achieve a lipid-lowering effect by lowering the levels of low-density lipoprotein. The results of this study found that, on the basis of the use of statins, the low-density lipoprotein decreased more significantly after the use of the C-117 formula, suggesting that the C-117 formula may strengthen the effect of statins.

The pathogenesis of AS is unclear, and the “endothelial injury” hypothesis is recognized by most people [21]. AS involves many biological processes, such as the inflammatory response, angiogenesis, and smooth muscle proliferation. Finally, lipids in the intima and proliferated and migrated smooth muscle cells form AS plaques [22]. AS plaques attach to the lumen of the artery, obstruct blood flow, and cause vascular stenosis in severe cases. Because AS is a progressive disease, the current clinical treatment advocates long-term intervention for high-risk patients; controlling blood pressure, blood sugar, and other risk factors; delaying the process; and preventing serious cardiovascular and cerebrovascular diseases [6]. The results of this study did not statistically validate the efficacy of the C-117 formula in the treatment of vulnerable plaques, but we found that the carotid IMT and plaque area in the C-117 formula group were lower than those before treatment, and the degree of change was greater than that of the control group. We speculate that the compound may delay the accumulation of a plaque and have certain lipid-lowering effect, but the effect is relatively mild, and the best therapeutic effect was not achieved in 180 days of treatment.

The C-117 formula consists of four drugs,* Hirudo *(ShuiZhi),* Curcumae Zedoary *(EZhu),* Atractylodis Rhizoma *(CangZhu), and* Endothelium Corneum Gigeriae Galli *(JiNeiJin), which contains two animal drugs. Hirudo's active ingredient, hirudin, protects endothelial cells by inhibiting thrombin activity, lowering lipids, and regulating the NO/endothelin balance. Hirudin can also inhibit the release of inflammatory factors, the formation of foam cells, and the proliferation of vascular smooth muscle cells [10, 11].* Curcumae Zedoary's* main component, curcumin, also inhibits endothelial cell proliferation and migration, so the combination of* Curcumae Zedoary* and* Hirudo* can reduce the occurrence of endothelial damage. In addition,* Curcumae Zedoary* can control the risk factors of AS by reducing the local inflammatory response, lower lipid levels, and inhibiting platelet aggregation [23, 24].* Atractylodis Rhizoma* could control blood pressure by inhibiting the activity of the angiotensin-inhibiting enzyme. it also could inhibits gastric acid secretion, Hypoglycemic and so on [25].* Endothelium Corneum Gigeriae Galli* is the inner wall of the dry sac of the* Gallus gallus domesticus* Brisson and contains a variety of active proteases, amino acids, and polysaccharides from* Endothelium Corneum Gigeriae Galli*.* Endothelium Corneum Gigeriae Galli* can delay the AS process by antioxidation and improving blood sugar and blood lipid levels and blood rheology parameters [12, 13]. Therefore, we speculate that the combination of the four drugs can help control blood lipids and blood pressure by inhibiting endothelial cells, inhibiting foam cell formation, inhibiting smooth muscle cell proliferation, antioxidation, and improving blood viscosity to delay the development of AS.

The limitations of this study include the following: (1) An insufficient sample size. This study included a total of 120 patients, and the small sample size limited the credibility and extrapolation of the analysis results. (2) A short research period, the intervention time of this study was too short to realistically reflect the efficacy and safety of the C-117. (3) The diagnostic method of CAS-vulnerable plaques is determination of the carotid artery colour by Doppler ultrasound, and the result is subjectively influenced by the operator. Although we conducted unified training in colour ultrasound examination for doctors before the experiment, we cannot eliminate the existence of subjective deviations. Therefore, there is a certain influence on the judgment of the results. Our follow-up research needs to use more objective examination methods for diagnosis and evaluation.

In summary, the C-117 formula may delay the AS process, but this study is limited by the sample size and study period. The results of this study failed to statistically verify this inference. However, we found that the C-117 formula has a mild effect in delaying the accumulation of plaques and lipid lowering. Clinical trials with large sample sizes, long-term interventions, and follow-up are needed to investigate this effect.

## Figures and Tables

**Table 1:** Study inclusion and exclusion criteria.

Inclusion criteria	
1	Meet the diagnostic criteria of carotid artery atherosclerosis
2	Ultrasound of carotid artery atherosclerotic plaque specifies it as an
	unstable plaque (including malakoplakia and mixed plaque);
3	Can persist on long-term medication
4	Older than 18 and under 80 years old
5	Meet the diagnostic criteria of hyperlipidaemia: Fasting serum total cholesterol ≥ 5.72 mmol/L or triglycerides ≥ 1.70 mmol/L or low density lipoprotein ≥ 1.8 mmol/L;

Exclusion Criteria:	

1	mRS rating higher than or equal to 3 before joining the trial group;
2	Carotid artery stenosis higher than or equal to 50%;
3	Confirmed or suspected vasculitis;
4	Infection, tumour or insufficient heart, liver and kidney functions
	(renal function higher than twice the upper limit in normal limits,
	HYHA heart function grading higher than or equal to level 2);
5	Acute myocardial infarction and unstable angina pectoris;
6	Acute cerebral arterial thrombosis;
7	Severe stenosis or occlusion of distal intracranial vessels;
8	Pregnant women or lactating mothers;
9	Known history of allergy to the trial drugs.

**Table 2:** Baseline characteristics.

	C-117 formula group	Placebo group
	(n=59)	(n=59)
Basic Features		

Age (Yr) $\overset{-}{X}\pm S$	64.76 ± 7.079	63.5 ± 7.691
≥60, n (%)	46(78.0%)	43(72.9%)
Gender		
Female	34(57.6%)	28(47.5%)
Male	25(42.4%)	31(52.5%)
Height (cm) $\overset{-}{X}\pm S$	161.39 ± 7.8	162.46 ± 6.18
Weight (kg) $\overset{-}{X}\pm S$	62.92 ± 11.0	62.13 ± 8.58
Systolic blood pressure, $\overset{-}{X}\pm S$, (mmHg)	126.29 ± 11.33	127.56 ± 13.85
Diastolic blood pressure, $\overset{-}{X}\pm S$, (mmHg)	71.88 ± 8.81	74.32 ± 11.37

Past history, n (%)		

Ischemic stroke or TIA	8(13.6%)	9(15.3%)
Diabetes	14(23.7%)	13(22.0%)
Hypertension	30(50.8%)	37(62.7%)
Hyperuricemia	6(10.2%)	5(8.5%)
Other medical history	30(50.8%)	31(52.5%)

Personal life history, n (%)		

Exercise		
Often	33(55.9%)	36(61.0%)
Occasionally	26(44.1%)	22(37.3%)
Never	0(0%)	1(1.75%)
Eat vegetables every day	55(93.2%)	52(88.1%)
Eat fruit every day	35(59.3%)	34(57.6%)
Smoking history	7(11.9%)	9(15.3%)
Drinking history	19(32.2%)	11(18.6%)

Taking lipid-lowering drugs, n (%)		

Atorvastatin calcium tablets	48(81.4%)	46(78.0%)
Other lipid-lowering drugs	10(16.9%)	10(16.9%)
Not taken	1(1.7%)	3(5.1%)

Combination therapy, n (%)		

Antihypertensive drugs	28(47.5%)	30(50.8%)
Hypoglycaemic agents	10(16.9%)	11(18.6%)
Antiplatelet drug	12(20.3%)	9(15.3%)
Other medication	20(33.9%)	12(20.3%)

**Table 3:** Plaque characteristics of the two groups.

	C-117 formula group (n=59)	Placebo group (n=59)	Z/T	P
*Bilateral IMT (mm) *				

Before treatment	1.88 ± 0.44	1.89 ± 0.41		
After treatment	1.78 ± 0.35*∗*	1.77 ± 0.33*∗*	-0.005	0.996

*Crouse score (mm)*				

Before treatment	4.77 ± 3.06	3.96 ± 2.60		
After treatment	3.96 ± 2.60*∗*	3.80 ± 2.91	-1.233	0.217

*Plaque area (mm* ^*2*^)				

Before treatment	56.07 ± 71.15	38.13 ± 29.86		
After treatment	53.92 ± 55.58	38.48 ± 46.60	-1.416	0.157

*Number of vulnerable plaques (n) *				

Before treatment	2.12 ± 1.22	1.80 ± 0.92		
After treatment	2.19 ± 1.27	1.93 ± 1.11	-0.960	0.337

*Number of stable plaques (n) *				

Before treatment	0.33 ± 0.54	0.25 ± 0.60		
After treatment	0.31 ± 0.56	0.24 ± 0.50	-0.673	0.501

*Total number of plaques (n) *				

Before treatment	2.46 ± 1.30	2.05 ± 1.11		
After treatment	2.49 ± 1.36	2.17 ± 1.25	-1.295	0.195

M, mean ± standard deviation. P is the P value of the FAS dataset;

*∗*P<0.05 vs. before treatment.

**Table 4:** Blood lipid levels of the two groups who had taken Lipitor.

	*C-117 formula group (n=48) *	*Placebo group (n=46) *	Z/T	P
*Lipid levels*				
*Triglyceride (mmol/L)*				

Before treatment	1.33 ± 0.57	1.57 ± 0.89		
After treatment	1.27 ± 0.58	1.30 ± 0.61	-0.983	0.329

*Total cholesterol (mmol/L)*				

Before treatment	5.02 ± 0.91	4.98 ± 0.86		
After treatment	4.65 ± 1.02*∗*	4.87 ± 0.95	-0.309	0.757

*Low-density lipoprotein (mmol/L)*				

Before treatment	3.06 ± 0.79	3.11 ± 0.80		
After treatment	2.89 ± 0.86	3.23 ± 0.81	-1.826	0.720

M, mean ± standard deviation. P is the P value of the FAS dataset;

*∗*P<0.05 vs. before treatment.

**Table 5:** Taking lipid-lowering drugs, n (%).

	C-117 formula group (n=59)	Placebo group (n=59)
Atorvastatin calcium tablets (Lipitor)	44(74.6%)	42(71.2%)
Other lipid-lowering drugs	7(11.9%)	9(15.3%)
Not taken or the lipid-lowering medication time < 20% of the 6-month treatment	8(13.5%)	8(13.5%)

**Table 6:** Blood lipid levels of the two groups whose Lipitor medication time *⩾*20% of the 6-month treatment.

	*C-117 formula group (n=44) *	*Placebo group (n=42) *	Z/T	P
*Triglyceride (mmol/L)*				

Before treatment	1.33 ± 0.58	1.56 ± 0.90		
After treatment	1.25 ± 0.53	1.27 ± 0.56*∗*	-0.430	0.966

*Total cholesterol (mmol/L)*				

Before treatment	5.02 ± 0.94	5.01 ± 0.78		
After treatment	4.62 ± 0.96*∗*	4.74 ± 0.85*∗*	-0.567	0.572

*Low-density lipoprotein (mmol/L)*				

Before treatment	3.09 ± 0.81	3.13 ± 0.78		
After treatment	2.86 ± 0.79*∗*	3.04 ± 0.72	-1.116	0.267

M, mean ± standard deviation. P is the P value of the FAS dataset;

*∗*P<0.05 vs. before treatment.

**Table 7:** Bilirubin levels of the two groups.

	*C-117 formula group (n=59) *	*Placebo group (n=59) *	Z/T	P
*Total bilirubin (umol/L)*				

Before treatment	10.74 ± 5.58	9.30 ± 2.64		
After treatment	11.08 ± 4.17	9.60 ± 2.32	-1.639	0.101

*Direct bilirubin (umol/L)*				

Before treatment	3.54 ± 1.56	3.20 ± 0.87		
After treatment	3.90 ± 1.24*∗*	3.54 ± 0.84*∗*	-1.561	0.119

*Indirect bilirubin (umol/L)*				

Before treatment	7.16 ± 4.17	6.06 ± 1.87		
After treatment	7.16 ± 3.10	6.06 ± 1.79	-1.587	0.112

M, mean ± standard deviation. P is the P value of the FAS dataset;

*∗*P<0.05 vs. before treatment.

## Data Availability

The data used to support the findings of this study are available from the corresponding author upon request.

## Abbreviations

C-117 formula: The Herbal Medicine C-117 formula
IMT: Intima-media thickness
CAS: Carotid atherosclerosis
AS: Atherosclerosis
KYMCRGOHTCM: The Key Unit of Methodology in Clinical Research of Guangdong Province Hospital of Traditional Chinese Medicine
GMP: Good Manufacturing Practices.

## References

[1] Feigin V. L., Nguyen G., Cercy K. (2018). Regional, and country-specific lifetime risks of stroke, 1990 and 2016. *The New England Journal of Medicine*.

[2] Longde W., Jianmin L., Yang Y. (2019). The prevention and treatment of stroke still face huge challenges ——brief report on stroke prevention and treatment in China, 2018. *Chinese Circulation Journal*.

[3] Wang W., Jiang B., Sun H. (2017). Prevalence, incidence and mortality of stroke in china: results from a nationwide population-based survey of 480,687 adults. *Circulation*.

[4] Zhao X., Hippe D., Li R. (2017). Prevalence and characteristics of carotid artery high-risk atherosclerotic plaques in chinese patients with cerebrovascular symptoms: a chinese atherosclerosis risk evaluation II study. *Journal of the American Heart Association*.

[5] Naylor A. R., Ricco J.-B., de Borst G. J., Halliday A. (2018). Management of atherosclerotic carotid and vertebral artery disease, 2017, clinical practice guidelines of the European society for vascular surgery (ESVS). *European Journal of Vascular and Endovascular Surgery*.

[6] AACE (2017). American association of clinical endocrinologists and american college of endocrinology guidelines for management of dyslipidemia and prevention of atherosclerosis, Version I. *AACE Clinical Case Reports*.

[7] Amarenco P. (2006). High-dose atorvastatin after stroke or transient ischemic attack. *Journal of Vascular Surgery*.

[8] Meiling G., Zhendong Z., Zhigang H. (2015). A reappraisal for the risks of statin therapy. *Chinese Journal of Arteriosclerosis*.

[9] Dormuth C. R., Hemmelgarn B. R., Paterson J. M. (2013). Use of high potency statins and rates of admission for acute kidney injury: multicenter, retrospective observational analysis of administrative databases. *BMJ*.

[10] Zhang E., Lixu X., Tongde Z. (2017). The research progress of hirudo on the related cells in the progression of atherosclerosis. *Chinese Journal of Arteriosclerosis*.

[11] Liu X., Gao M.-F., Kong Y. (2017). Bioactive constituents and pharmacological effects of leech. *Chinese Journal of Pharmaceutical Biotechnology*.

[12] Changxing J., Dingyun J., Qingping X. (2012). Effects of JiNeiJin's astragalosides on blood lipids, hemorheology, and oxidative stress markers in hyperlipidemic rats. *Pharmacology and Clinics of Traditional Chinese Medicine*.

[13] Guo X., Jiguang F., Kejie H. (2000). Experimental study on lipid-lowering, anticoagulant and hemorheological effects of JiNeiJin. *Information on Traditional Chinese Medicine*.

[14] Rongming L. (2016). *A prospective study to evaluate the safety and efficacy treated with Chinese insects and herbal medicine formula for patients with vulnerable plaques of carotid artery [Master, thesis]*.

[15] Chinese Medical Association Neurology Branch (2015). Guidelines for secondary prevention of ischemic stroke/transient ischemic stroke in China 2014. *Chinese Journal of Neurology*.

[16] Chinese Medical Association Ultrasound Medical Branch (2015). Carotid ultrasound examination specification for healthy physical examination population. *Chinese Journal of Health Management*.

[17] Hirayama A., Saito S., Ueda Y. (2011). Plaque-stabilizing effect of atorvastatin is stronger for plaques evaluated as more unstable by angioscopy and intravenous ultrasound. *Circulation Journal*.

[18] Erdogan D., Gullu H., Yildirim E. (2006). Low serum bilirubin levels are independently and inversely related to impaired flow-mediated vasodilation and increased carotid intima-media thickness in both men and women. *Atherosclerosis*.

[19] Novotný L., Vítek L. (2003). Inverse relationship between serum bilirubin and atherosclerosis in men: a meta-analysis of published studies. *Experimental Biology and Medicine*.

[20] Vítek L. (2017). Bilirubin and atherosclerotic diseases. *Physiological Research*.

[21] Ross R. (1995). Cell biology of atherosclerosi. *Annual Review of Physiology*.

[22] Lusis A. J. (2000). Atherosclerosis. *Nature*.

[23] Cui Y.-Y., Liu J.-G., Zhao F.-H., Shi D.-Z. (2015). Advances in studies on pharmacological action of mainchemical constituent of curcumae zedoary in preventing in-stent restenosis. *China Journal of Chinese Materia Medica*.

[24] Qian W., Fuhai Z., Dazhuo S. (2012). Advances in cardiovascular pharmacology research of rhizoma curcumae and its extracts. *Chinese Journal of Integrated Traditional Chinese and Western Medicine*.

[25] Aiping D., Ying L., Zhitao W. (2016). Advances in studies on chemical compositions of Atractylodes lancea and their biological activities. *China Journal of Chinese Materia Medica*.

